# Fine Needle-Diathermy Regresses Pathological Corneal (Lymph)Angiogenesis and Promotes High-Risk Corneal Transplant Survival

**DOI:** 10.1038/s41598-018-24037-3

**Published:** 2018-04-09

**Authors:** Viet Nhat Hung Le, Ann-Charlott Schneider, Rebecca Scholz, Felix Bock, Claus Cursiefen

**Affiliations:** 1 Department of Ophthalmology, University Hospital of Cologne, Cologne, Germany; 2 Department of Ophthalmology, Hue University of Medicine and Pharmacy, Hue, Vietnam; 3 Center for Molecular Medicine Cologne (CMMC), University of Cologne, Cologne, Germany

## Abstract

Pathological corneal hem- and lymphangiogenesis are prime risk factors for corneal graft rejection. Fine needle-diathermy (FND) is an option to regress corneal blood vessels; however, whether this treatment besides clinically visible blood vessels also affects invisible lymphatic vessels is so far unknown. Here we test the hypothesis that FND destroys not only blood but also lymphatic vessels, thereby promotes corneal high-risk graft survival. The effect of FND was studied *in vivo* using BALB/c mice and the model of suture-induced corneal neovascularization. Mice were divided into three groups: FND, ANTI (anti-inflammatory therapy) and NON (control). Five, 7, 10 and 20 days after cauterization, corneas were harvested and stained with LYVE-1, CD31 to quantify (lymph)angiogenesis. The long-term survival of allografts was compared between the three groups. FND caused significant regression of both blood and lymphatic vessels compared to the control group at all time points (p < 0.05) with the most obvious effect at day 7 (p < 0.01). Graft survival was significantly prolonged when transplants were placed into the FND pretreated group (p < 0.0001). The effect of the anti-inflammatory therapy alone was less effective compared to FND (p < 0.05). This novel lymphangioregressive effect of FND can be used clinically to precondition high-risk recipients to promote graft survival.

## Introduction

The physiologically transparent and avascular cornea plays a crucial role in good vision by providing a proper anterior refractive surface and by allowing the entry of light^1,2^. The avascularity of the cornea is maintained amongst others by the expression of soluble forms of the three major vascular endothelial growth factor (VEGF) receptors and anti-angiogenic and anti-lymphangiogenic factors (for review see^1,2^). This status can be destroyed by the invasion of corneal neovascularization (CoNV) from the limbal vascular arcade into the cornea, caused by a wide variety of diseases^3^. CoNV can be helpful in the healing process to repair and regenerate some corneal insults, however, the presence of blood and lymphatic vessels after the healing process not only interferes with corneal clarity but also disrupts the “immune privileged” status of the cornea^4^. Efferent blood vessels as well as afferent lymphatic vessels associated with the regional lymph node are the three structural components of the immune reflex arc which have a major role in immune responses against foreign tissue, especially in graft rejection^5^. Recently, Dietrich *et al*. showed that lymphatic vessels are more important than blood vessels in immune rejection after corneal transplantation^6^. Therefore, it is important to find methods to block and regress both pathological corneal angiogenesis and lymphangiogenesis.

Many different techniques have already been suggested to regress corneal blood vessels. Topical corticosteroids were first used about 60 years ago to markedly reduce corneal vascularization^7^. Although its effect in suppressing blood and lymphatic vessels has been so far successfully tested^8–11^, the long-term use of topical steroid leading to serious side effects (cataract, glaucoma, superinfection, herpes simplex recurrence) has still been a limiting aspect. An antiangiogenic antisense oligonucleotide, GS101, has been shown to significantly inhibit corneal blood vessels in phase II and III trials^12,13^. Other treatments for corneal neovascularization include argon laser, photodynamic therapy and 577 mm yellow dye laser^3,14–19^. However, their variable and temporary effect, their lack of availability, high cost, inaccessibility to ophthalmologists and their complications require the need for a new simple, effective and safe alternative treatment. Recently, anti-VEGF therapy has been demonstrated to be effective in attenuating corneal angiogenesis and lymphangiogenesis^20,21^. Nevertheless, isolated anti-VEGFs seem to be ineffective in regressing established, mature vessels which depend less on angiogenic growth factors^22^.

Fine needle diathermy (FND) is currently one clinical choice for managing mature pathologic corneal blood vessels. This technique was first described and clinically utilized by Pillai *et al*.^23^. CoNV was precisely occluded by using 10-0 monofilament black nylon suture and diathermy current applied to the needle being located in the stromal vessel. This approach has shown potential also e.g. in the context of high-risk keratoplasty patients^23^.The long-term efficacy and safety of this therapy were documented and demonstrated by several further researches^24–29^. FND is now widely used because of its effectiveness, safety, simple procedure, and inexpensive equipment which make it accessible to any eye unit throughout the world. However, whether this treatment besides clinically visible blood vessels also affects lymphatic vessels is so far unknown. Furthermore, formal trials testing its effect on corneal graft survival in the high-risk scenario are missing. Here we test the hypothesis that fine needle diathermy destroys not only visible blood but also invisible lymphatic vessels and thereby prolongs the survival grafts of high-risk corneal transplantation.

## Results

### FND regresses mature corneal blood and lymphatic vessels

**Figure 1:**
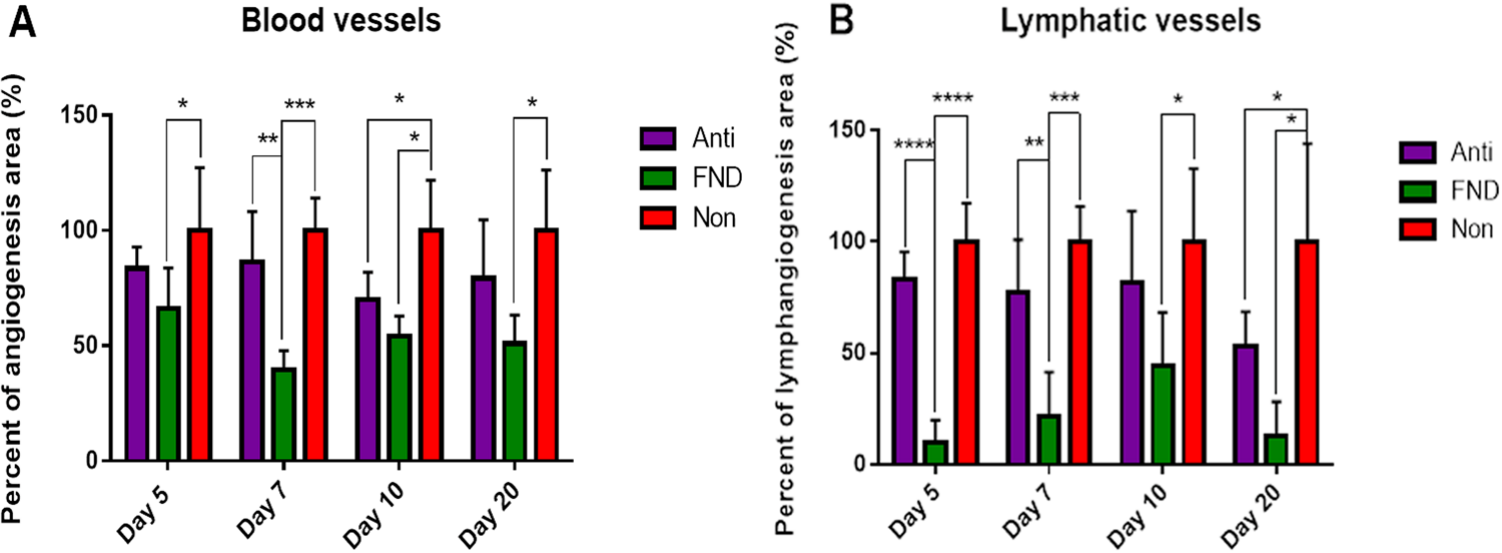
Effect of FND on regression of blood vessels (**A**) and lymphatic vessels (**B**) at four time points after treatment (after 14 days of corneal suture placement). Compared to the non-treated group (NON: red column), the FND treated group (green column) resulted in significant regression of both blood and lymphatic vessels at all four time points (p < 0.05). The most obvious effect of FND is observable at day 7 with the reduction of hemangiogenesis by 60% (p < 0.0001) and lymphangiogenesis by approximately 80% (p < 0.0001) as compared to the NON group.

**Figure 2:**
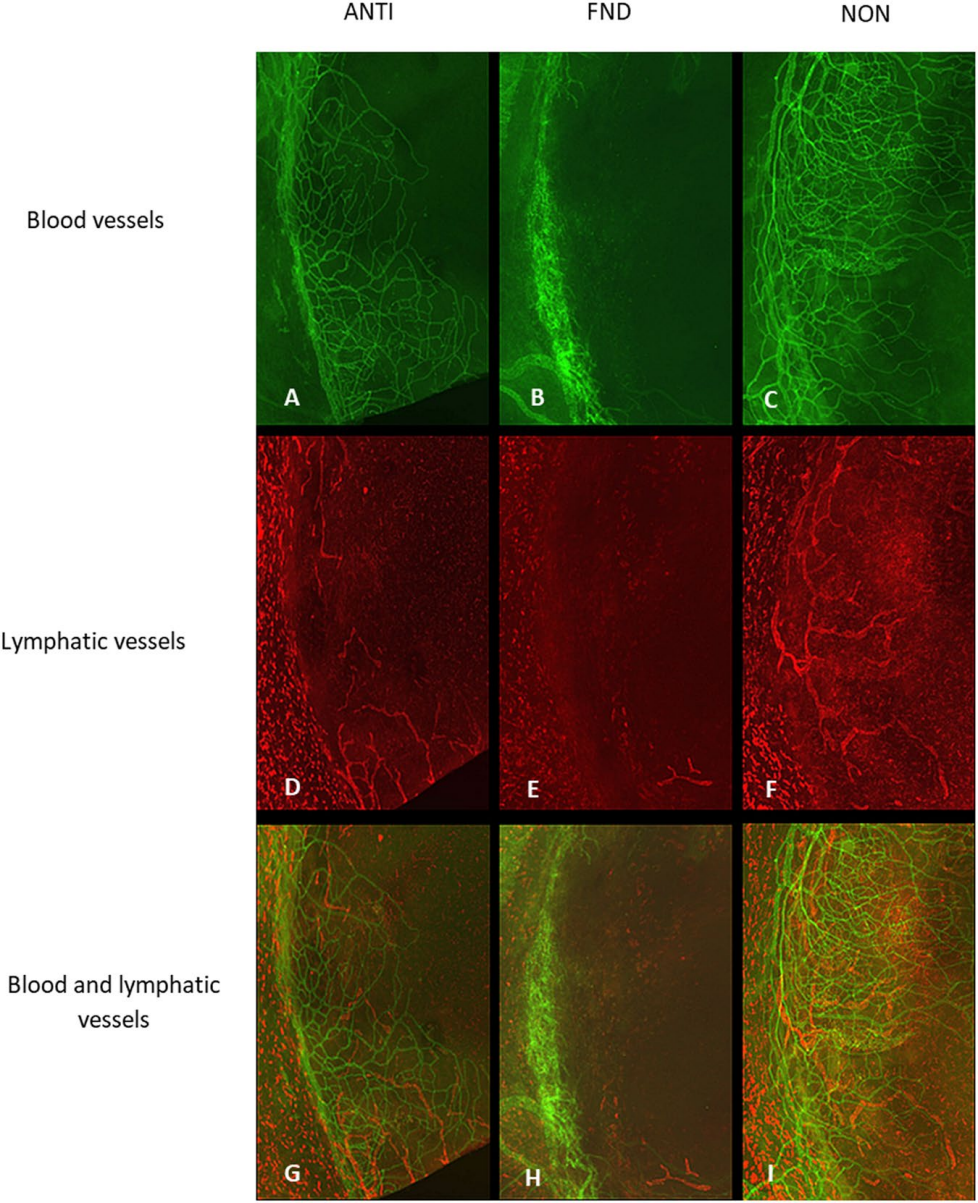
Corneal whole mounts were stained with CD31 for blood vessels and LYVE-1 for lymphatic vessels. Immunohistochemistry pictures at day seven after FND treatment showing FND treated corneas with significantly reduced areas of blood (**B**) and lymphatic vessels (**E**) as compared to controls. (**A**–**C**) blood vessels; (**D**–**F**) lymphatic vessels; (**G**–**I**) blood and lymphatic vessels merged.

To identify the effect of FND on mature pathologic corneal blood and lymphatic vessels and to determine the best time point of FND treatment, corneas of high-risk eyes after 14 days of suture placement were treated and then harvested subsequently five, 7, 10 and 20 days after FND. Quantitative analysis of the neovascularised corneal area identified by immunohistochemical staining revealed that both blood and lymphatic vessels were significantly reduced in the FND group compared to the NON group at all time points (p < 0.05). The most significant effect of FND on regression of both blood and lymphatic vessels was observable at day 7 after FND treatment compared to the NON and ANTIgroups (Figs 1 and 2).

**Figure 3:**
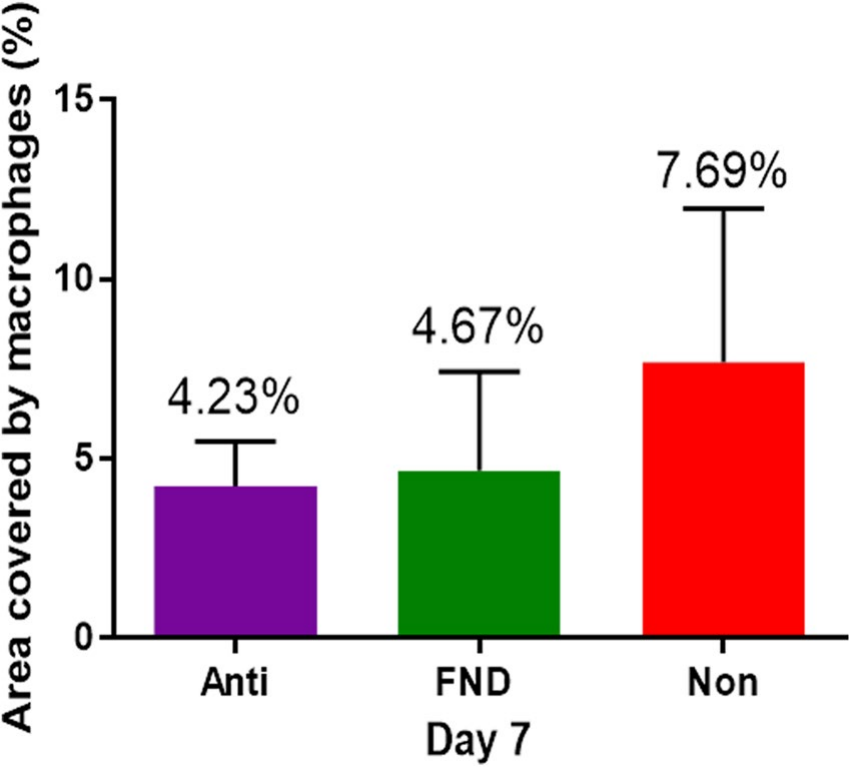
The corneal area covered by LYVE-1 positive macrophages after different pretreatments. Although the LYVE-1 (+) cell infiltration at day seven in the NON group (7.69 ± 4.28) was increased in relation to the ANTI and FND groups (4.23 ± 1.25 and 4.67 ± 2.76 respectively), there was no significant difference between these groups.

Anti-inflammatory eye drops (Dexamethasone) were applied topically to reduce the additional angiogenic stimulus caused by the FND procedure itself. Indeed, the results of the corneal area covered by LYVE-1 positive macrophages at day seven showed the similarity of cell infiltration between the ANTI and FND groups (4.23 ± 1.25 and 4.67 ± 2.76 respectively) (Fig. 3).

### FND pre-treatment promotes graft survival after subsequent transplantation into high-risk recipient beds

**Figure 4:**
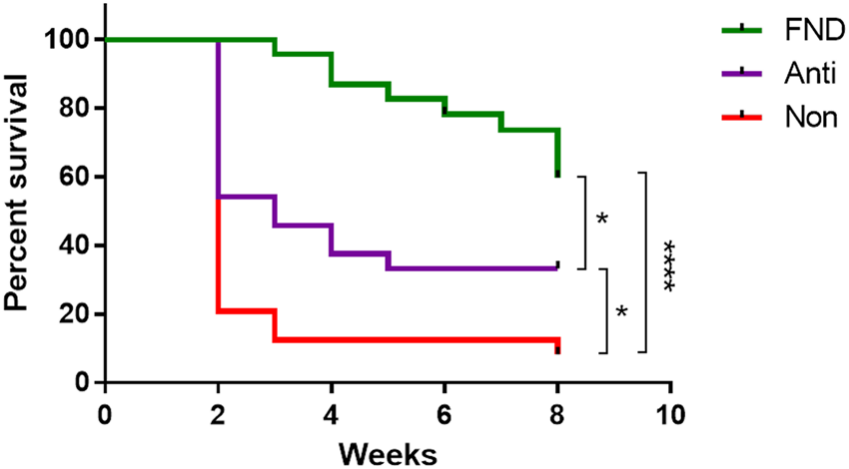
Preoperative fine needle diathermy of pathologic corneal blood and lymphatic vessels in vascularized high-risk eyes promotes graft survival after subsequent allogenic high-risk corneal transplantation. Two weeks after corneal suture placement, mice were treated with fine needle diathermy and anti-inflammatory drops (FND; green line) or with anti-inflammatory eye drops only (ANTI; purple line). Graft survival was compared to a control group which did not receive cautery or anti-inflammatory therapy (NON; red line). The graft survival was significantly prolonged when transplants were placed into recipient bed of pretreated groups. (FND versus NON: 60.9% vs. 8.3%, p < 0.0001; ANTI versus NON: 33.3% vs. 8.3%, p = 0.0164). Between two treated groups, our results showed that FND treatment significantly improved the graft survival in comparison to isolated anti-inflammatory therapy (FND versus ANTI: 60.9% vs. 33.3%, p < 0.05).

After determining the best time point of the (lymph)angio-regressive effect of FND, we assessed whether the preoperative regression of blood and lymphatic vessels in high-risk recipient beds by using FND could improve the graft survival after subsequent transplantation. The long-term survival of C57BL/6 donor buttons transplanted into highly vascularized BALB/C recipient beds was compared between mice receiving an intrastromal FND and anti-inflammatory eye drops (FND group) and those receiving only anti-inflammatory drops (ANTI group) or those receiving no further treatment (NON group). We could demonstrate that high-risk mice pre-treated with FND and anti-inflammatory eye drops showed a significantly improved long-term graft survival at 8 weeks after transplantation (60.9%), compared to those in eyes of ANTI (33.3%, p = 0.0108) and NON group (8.3%, p < 0.0001, n = 24 mice in each group) (Fig. 4).

## Discussion

The experiments presented herein allow four important conclusions to be drawn:

(i) Fine needle diathermy, besides clinically visible blood vessels, also destroys clinically invisible lymphatic vessels.
(ii) Since lymphatic vessels are clinically not visible, the approach of circular perilimbal FND as used herein nearly completely regresses lymphatic vessels in pathologically prevascularized high-risk eyes.
(iii) Pretreatment of pathologically prevascularized high-risk eyes with FND and anti-inflammatory eye drops significantly improves graft survival after subsequent transplantation and opens a clinically realistic treatment option for improvement of graft survival in high-risk eyes.
(iv) In fact, this to our knowledge is the first evidence that preconditioning of high-risk eyes by induced regression of pathologic corneal blood and lymphatic vessels in these eyes prior to transplantation promotes subsequent graft survival.

Corneal neovascularization (CoNV) threatens corneal transparency, visual acuity and greatly increases the risk of graft rejection^30^. Especially the regression of already established blood and lymphatic vessels is one of the major challenges for ophthalmologists. The fine needle diathermy technique, first described by Pillai *et al*.^23^, could be proven to be effective and safe in blood vessel regression by several studies thereafter^24–26^. However, whether this treatment besides clinically visible blood vessels also affects lymphatic vessels was so far unknown.

Our findings demonstrate for the first time the effect of FND on regression of lymphatic vessels in the cornea *in vivo*. Since lymphatic vessels are not visible at the slit-lamp and seem to be the primary mediator of immune reactions in vascularized high-risk eyes, imaging clinically invisible corneal lymphatics is even more challenging and important than imaging corneal blood vessels. Here a great unmet need exists in the clinic to visualize human corneal lymphatic vessels not identifiable using slit-lamp magnification. To overcome this, in fact, we just presented two novel approaches to detect these lymphatic vessels. A novel, easy to use and low priced method to image corneal lymphatic vessels using commercially available fluorescein is to inject fluorescein dye into the corneal stroma and to image lymphatics using commercially available angiography devices otherwise used for retinal angiography (e.g. HRA)^31^. By using this novel method, we can principally selectively destroy corneal blood and/or lymphatic vessels and thereby decrease the amount of tissue destruction caused by fine needle diathermy. Furthermore, it was recently published that high-resolution OCT imaging allows for non-contact imaging of pathologic corneal lymphatic vessels thereby also enabling a more selective and tissue-sparing destruction of corneal lymphatic vessels in patients in the future^32^. However, in this study, we used the suture-induced corneal neovascularization assay in the murine model which caused strong circumferential neovascularization. Therefore, the circular perilimbal approach used in this study (FND for 360 degrees along the marginal corneal vascular arcade) is a realistic option to nearly completely regress all corneal lymphatic vessels. When used alone, FND can also be disadvantageous. It may, for example, induce the release of proangiogenic factors, especially when applied to multiple vessels^23^. Hence, in 2015 Spiteri *et al*. suggested the use of corneal angiography to identify and selectively close the afferent vessels and thereby minimize tissue destruction without altering the effectiveness of FND^33^. Indeed, Koenig^27^, Elbaz^28^ and Hussain^29^ proposed combined FND and topical or subconjunctival Bevacizumab (Avastin®) to compensate for the limitation of the other. In our experiments, we combined FND with anti-inflammatory eye drops (dexamethasone) to reduce the additional angiogenic stimulus caused by the FND procedure itself. Generally, the effect of topically instilled dexamethasone on hemangiogenesis and lymphangiogenesis is well known from clinical trial and previous experimental studies (Hos *et al*.^11^, Kim *et al*.^8^ and Mirabelli *et al*.^9^). In these studies, topical dexamethasone application resulted in the regression of angiogenesis by 57–93.1% and lymphangiogenesis by 55% at day seven. In our study, blood and lymphatic vessels were significantly suppressed by corticosteroid treatment by 30% at day ten and by 47% at day twenty respectively. The difference between these studies may be because of different animals studied, different neovascularization assays and different methods of morphometry of vascularized corneal areas applied. The best effect of combined treatment of FND with dexamethasone, by contrast, in our study was observed at day seven with the reduction of hemangiogenesis by 60% (p < 0.0001) and lymphangiogenesis by approximately 80% (p < 0.0001) compared to controls. In comparison with the group treated with only dexamethasone, the combined treated group had significantly stronger inhibitory effects on hem- and lymphangiogenesis *in vivo* (p < 0.01). These results suggest that fine needle diathermy combined with topical dexamethasone seems to be an effective treatment to occlude not only blood vessels but also clinical invisible lymphatic vessels.

In patients, placement of grafts into an avascular and non-inflamed bed in the so-called “normal-risk” scenario carries a 2-year survival of approximately 90%^34,35^. Graft success rate, on the contrary, falls to 10% if the corneal recipient bed is inflamed and highly vascularized. In the mouse model, corneal allograft survival is reduced from 50% after six weeks in normal-risk transplantation to nearly 0% after two weeks in high-risk recipient bed^36,37^. Our experiments showed similar results of graft survival in the control (NON) group (8.3% after 8 weeks sustained follow-up). Due to the effectiveness of FND on the regression of both corneal blood and lymphatic vessels (both arms of an immune reflex arc), the long-term high-risk corneal graft survival was significantly improved compared to controls (p < 0.0001) and isolated anti-inflammatory therapy (p < 0.05). The graft survival in ANTI group was significantly higher than that in NON group at the end of the experiment (p < 0.05). Kim *et al*. also indicated that corticosteroid pretreatment is effective to improve graft survival in high-risk penetrating keratoplasty^8^. The difference between this and our study is that in study of Kim *et al*. corticosteroid was injected into subconjunctival space two weeks before penetrating keratoplasty and continued for four weeks after the surgery. We applied corticosteroids topically only for one week after FND and discontinued after the corneal transplantation to avoid as much as possible the side effects of long-term usage of corticosteroids.

Taken together, our study shows that circular fine needle cautery combined with anti-inflammatory therapy can effectively destroy not only corneal blood but also clinically invisible lymphatic vessels in high-risk eyes and thereby significantly improves graft survival after subsequent corneal transplantation. This concept of lymphangioregressive pretreatment in vascularized HIGH-RISK EYES can be translated into the clinic and should next be tested in a clinical trial^2^.

## Materials and Methods

### Mice and anesthesia

All experimental procedures were approved by the local animal care and use committee (Landesamt fuer Natur, Umwelt und Verbraucherschutz Nordrhein-Westfalen; AZ 84-02.04.2016.A055) and conformed to the Association for Research in Vision and Ophthalmology’s Statement for the Use of Animals in Ophthalmology and Vision Research. Female BALB/C mice, purchased from Charles River Laboratories, Sulzfeld, Germany (aged 6–8 weeks) were used in the mouse model of suture-induced corneal inflammation and neovascularization assay^31^. For the corneal transplantation, six-to eight-week-old female C57BL/6 mice (Charles River Laboratories, Sulzfeld, Germany) were used as donors and same-aged female BALB/C mice as recipients. Prior to surgery, mice were deeply anesthetized with an intraperitoneal injection of a mixture of Ketanest (8 mg/kg) and Rompun (0.1 ml/kg).

### Suture-induced corneal inflammation and neovascularization assay

The mouse model of suture-induced inflammatory neovascularization was performed as previously described^36,38,39^. Three 11-0 nylon sutures (Serag Wiessner, Naila, Germany) were placed intrastromally on the right eye with two stromal incursions extending over 120 degrees of corneal circumference each. The outer point of suture placement was chosen near the limbus and the inner suture point was placed near the center of cornea equidistant from the limbus to obtain standardized angiogenic responses. Sutures were left in place for 14 days and were then removed. After suture removal, study mice were divided into three groups: FND, ANTI and NON (5 mice per group) and complied with the protocols for each group mentioned below.

### Fine needle diathermy (FND) procedure

**Figure 5:**
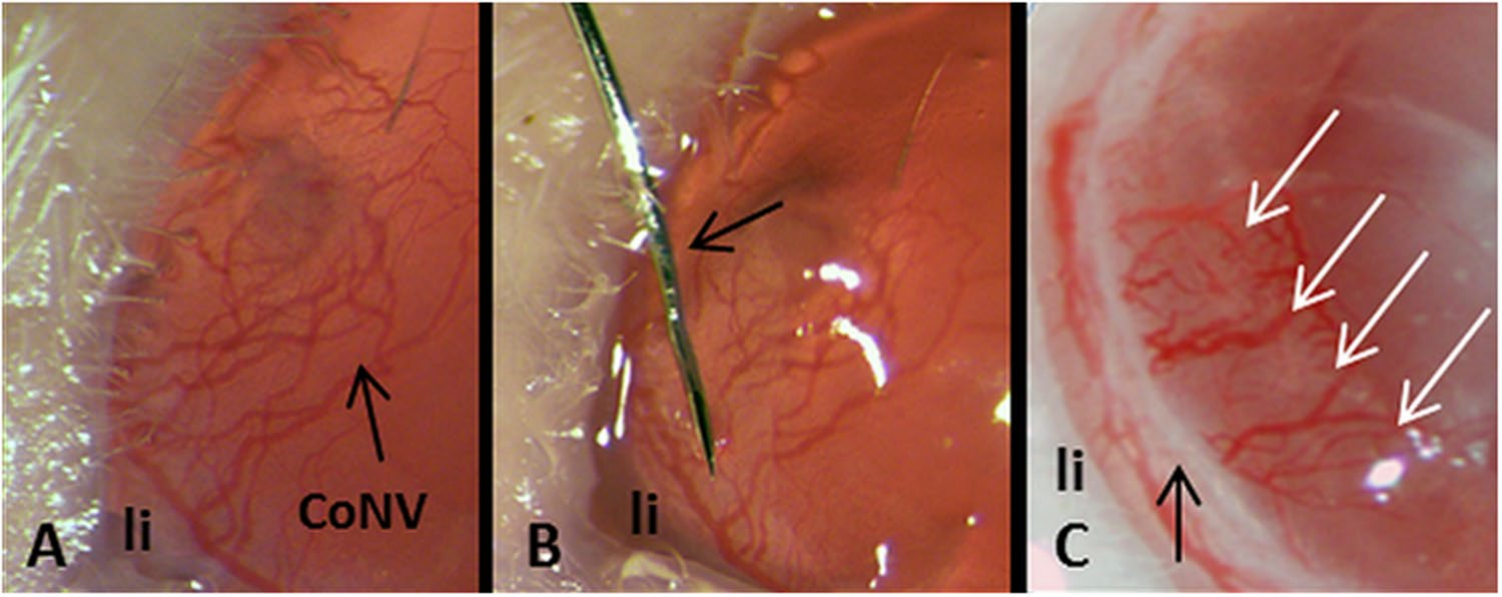
Fine needle diathermy as a pretreatment in vascularized high-risk recipient eyes with pathological corneal neovascularization (CoNV; (**A**)). (**B**) Fine needle (arrow) was placed intrastromally and parallel to limbus (li). (**C**) After FND treatment, coagulated tissue (black arrow) was seen as a white band, separating corneal limbus (li) and coagulated vessels without erythrocyte flow (white arrows).

FND was performed after removal of the suture under topical anesthesia with Conjuncain®EDO® (Bausch and Lomb, USA), by using non-alternating current from a unipolar diathermy unit also used clinically (VIO 50 C, Erbe Elektromedizin GmbH, Tübingen, Germany). A stainless steel 3/8 circle round-bodied, single-armed needle attached to an 11-0 nylon suture (Serag Wiessner, Naila, Germany) was used with a microsurgical needle holder (Geuder AG, Germany). The needle was inserted intrastromally close to and parallel to the corneal limbus. The power was set to 1 W and in the soft-coagulating mode, the fine angled needle (Ø 0.2 mm, L 40 mm, Erbe Elektromedizin GmbH, Tübingen, Germany) connected to the electrode handle, was brought into contact with the shaft of the surgical needle. The contact was maintained until mild blanching of the corneal stroma and/or corneal shrinkage occured (Fig. 5). This technique was repeated until reaching 360-degree circumferential cauterization. Post-operatively, to closely mimic the situation in the clinic, mice were treated with topical antibiotic and steroid combination treatment (Isopto Max, Alcon), five drops (5 µl/drop) per day for one week which was tapered off subsequently until the end of experiments.

Mice in ANTI group only received antibiotic and steroid combination treatment (Isopto Max) as topical drops with the same dosage and regimen compared with FND group, while the NON group did not obtain any anti-inflammatory treatment or FND after suture removal.

To determine the best time point of the effect of the FND treatment on regression of both corneal blood and lymphatic vessels, mice were euthanized and corneas were harvested five, 7, 10 and 20 days post-treatment. Whole-mount double-immunohistochemistry using CD31 and LYVE-1 was performed as described below.

### Corneal allogeneic transplantation in mice

Corneal allografting in the mouse model was performed as previously reported^6,40,41^. First, a button of the donor cornea of C57BL/6 was excised by marking the central 2 mm of the cornea using a 2.0 mm bore and cutting with curved Vannas scissors. The recipient BALB*/*C mouse was anesthetized, and the graft bed was prepared on the right eye by cutting out a circular 1.8 mm central area of the cornea. Subsequently, the graft was secured to the host corneal tissue with eight equidistant interrupted 11-0 nylon sutures (Serag Wiessner, Naila, Germany). Antibiotic ointment (Floxal, Bausch and Lomb, USA) was applied on the transplant and eyelids were sutured with an 8-0 suture (Serag Wiessner, Naila, Germany) to avoid damaging the graft. Tarsorrhaphy and corneal sutures were removed after seven days, and grafts were examined once a week until week eight after transplantation. The opacity of the grafts was evaluated and scored as previously described^40^. Clinical scores of corneal grafts for opacity were as follows: 0, clear; +1, minimal, superficial (nonstromal) opacity; pupil margin and iris vessels readily visible through the cornea; +2, minimal, deep (stromal) opacity; pupil margins and iris vessels visible; +3, moderate stromal opacity; only pupil margin visible; +4, intense stromal opacity; only a portion of pupil margin visible; and +5, maximum stromal opacity; anterior chamber not visible. Grafts with opacity scores greater than +2 were considered to be rejected. The survival experiment comprised three groups with 12 mice per group. All grafted eyes with infection, abnormal graft and loss of anterior chamber were excluded from further consideration.

### Immunohistochemistry and morphological analysis of corneal flat mounts

The blood and lymphatic vessels in corneal wholemounts were double stained as described previously^42–44^. Excised corneas were rinsed two times in PBS and then fixed in acetone for 30 minutes. After washing in PBS three times, and blocking with 2% bovine serum albumin (BSA) in PBS, the corneas were stained overnight (in dark, at 4 °C) with rabbit anti-mouse LYVE-1 (1:200; AngioBio, Del Mar, CA, USA) for lymphatic vessels and with a FITC-conjugated rat anti-mouse CD31 antibody (1:100; BD Pharmingen, BD Biosciences, USA) for blood vessels. On the next day, LYVE-1 was detected by using a goat-anti-rabbit Cy3-conjugated secondary antibody (1: 100; Dianova). Double stained wholemount images were assembled automatically from nine to 12 images taken at x100 magnification with a fluorescence microscope (BX53, Olympus Optical Co., Hamburg, Germany). Afterwards, the areas covered with blood and lymphatic vessels were detected with an algorithm established in the image analyzing program Cell^F (Olympus Soft Imaging Solutions GmbH, Münster, Germany), as previously described^39^. Briefly, before analysis, gray value images of the whole mount images were modified successively by four software-based filters. Afterwards, the total corneal area was outlined by using the innermost vessel of the limbal arcade as the border (region of interest; ROI), and the area covered by vessels defined by threshold setting was calculated (in mm^2^). The mean vascularized area of corneas in the NON group was defined as 100%, and the corneal vascularized areas in the other groups were then set into relation to this group. The area covered by LYVE 1 (+) cell was also quantified at day seven by using program Cell^F.

### Statistical analysis

Statistical analyses were performed with Microsoft Excel 2010 (Microsoft Corp., Redmond, WA) and Prism 6 version 6.07 (GraphPad Software, San Diego, California, USA). Statistical significance was determined using the one-way ANOVA test. P < 0.05 was considered statistically significant. Postoperative survival of the corneal allografts was analyzed using Kaplan–Meier survival curves with Logrank test. Graphs were drawn using Prism6.

### Data availability

The datasets generated during and analyzed during the current study are available from the corresponding author on reasonable request.

## Electronic supplementary material

Supplementary figure
