# Supplementary material for: Fine Needle-Diathermy Regresses Pathological Corneal (Lymph)Angiogenesis and Promotes High-Risk Corneal Transplant Survival
Source: Sci Rep. 2018 Apr 9;8:5707. doi: 10.1038/s41598-018-24037-3 (PMC5890271; doi:10.1038/s41598-018-24037-3)
Supplement: Supplementary file 1 — Supplementary figure [file 41598_2018_24037_MOESM1_ESM.pdf]

FINE NEEDLE-DIATHERMY REGRESSES PATHOLOGICAL CORNEAL (LYMPH)ANGIOGENESIS AND PROMOTES HIGH-RISK CORNEAL TRANSPLANT SURVIVAL

Viet Nhat Hung Le[1,2], Ann-Charlott Schneider[1], Rebecca Scholz[1], Felix Bock[1,3,*] and Claus Cursiefen[1,3]

1 Department of Ophthalmology, University Hospital of Cologne, Germany

2 Department of Ophthalmology, Hue University of Medicine and Pharmacy, Vietnam

3 Center for Molecular Medicine Cologne (CMMC), University of Cologne, Germany

*Correspondence: Felix Bock, PhD, Department of Ophthalmology, University of Cologne, Kerpener Strasse 62, 50924 Cologne, Germany; phone: +49 221 478 -97789; fax: -5094; felix.bock@uk-koeln.de; www.augenklinik.uk-koeln.de

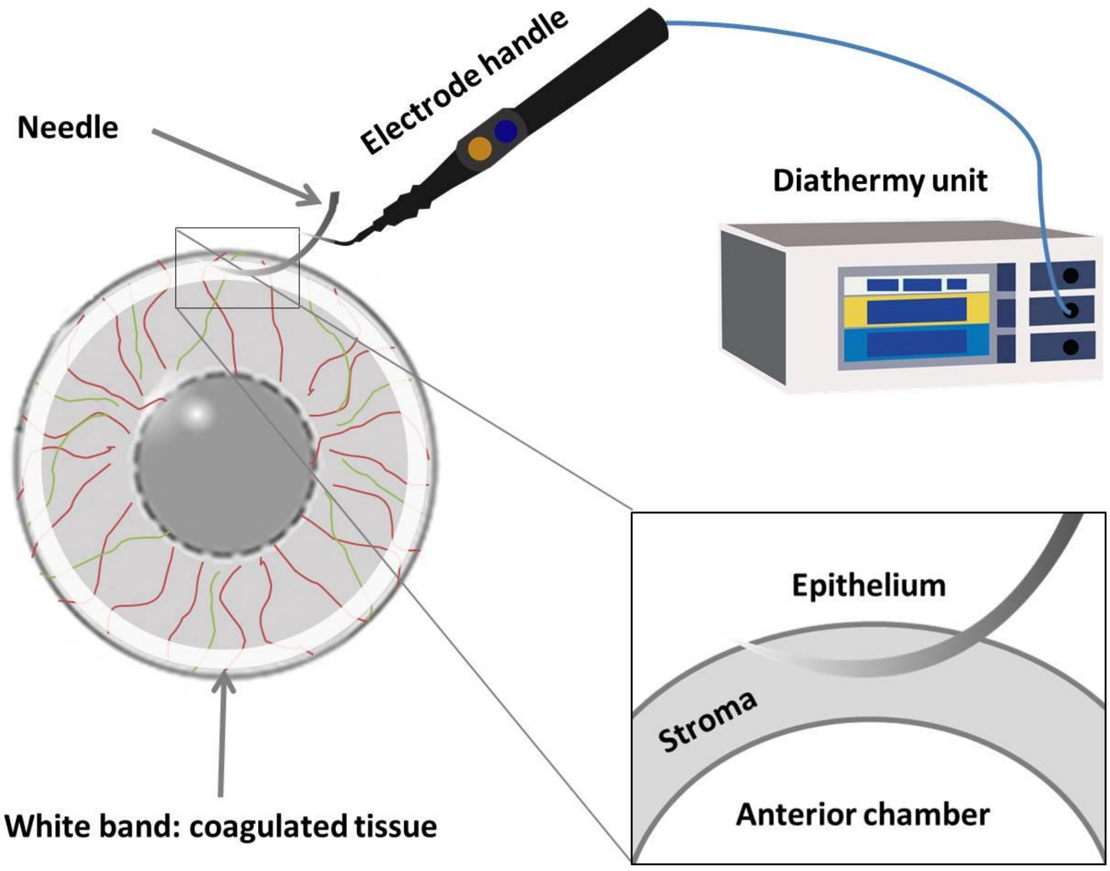

Supplementary figure: A circular fine-needle cautery approach was used in a 360 degree fashion to induce a small band of coagulated stromal tissue to regress both pathologic corneal blood as well as lymphatic vessels.
